# Effects of cigarette smoking on the respiratory epithelium and its role in the pathogenesis of chronic rhinosinusitis

**DOI:** 10.1016/S1808-8694(15)30557-7

**Published:** 2015-10-19

**Authors:** Edwin Tamashiro, Noam A. Cohen, James N. Palmer, Wilma Terezinha Anselmo Lima

**Affiliations:** 1 PhD. Attending Physician. Faculdade de Medicina de Ribeirão Preto da Universidade de São Paulo; 2 PhD, Professor. Faculdade de Medicina de Ribeirão Preto da Universidade de São Paulo; 3 PhD, Professor. Faculdade de Medicina de Ribeirão Preto da Universidade de São Paulo; 4 Associate Professor. Faculdade de Medicina de Ribeirão Preto da Universidade de São Paulo

**Keywords:** respiratory mucosa, sinusitis, tobacco

## Abstract

The increasing consumption of cigarettes has aroused concerns about the development and worsening of diseases, particularly those related to the respiratory tract.

**Aim:**

In this paper we review the evidence suggesting the effects of cigarette smoking on the respiratory epithelium and its role in the pathogenesis in chronic rhinosinusitis.

**Conclusions:**

Although there is evidence supporting a link between smoking and CRS, studies suggest that there might be individual susceptibility to cigarette smoking causing the development and/or maintenance of CRS. Proper patient educations to quit smoking as well as reinforcement of antismoking campaigns are extremely important to control this disease of major socio-economic impact.

## INTRODUCTION

Cigarette smoking is associated with the main current preventable cause of death, with growing importance especially in developing countries, such as Brazil. In its most recent publication on smoking, the World Health Organization pointed out that, among the eight main causes of death in the world today, six are associated with tobacco exposure, among them we stress inflammatory and infectious diseases of the respiratory tract.^1^ According to data from the Ministry of Health (2006), 16.2% of the adult Brazilian population smoke cigarettes daily.^2^ Despite the broad advertisement in the media about the harmful effects of cigarette smoking, very little is known about the association between cigarette exposure and the development of associated disorders such as chronic rhinosinusitis (CR). A better understanding of the physiopathogenic mechanisms involved in CR could lead to better treatment for these patients.^3^,^4^

## OBJECTIVES

In the present paper we reviewed the evidence pointing towards the cigarette smoke effects on the respiratory epithelium as well as its role on the pathophysiology of chronic rhinosinusitis.

## METHOD

Traditional asystematic review of papers indexed on the LILACS and MEDLINE databases from 1960 to 2009.

## LITERATURE REVIEW

1) Cigarette smoking effects on the respiratory epithelium

The epithelium coating the upper respiratory tract acts as a first line of defense against invasive agents (pollutants, allergens, microorganisms), and it can cause upper airway symptoms and diseases when in contact with these agents.^5^

Inhaled cigarette smoke, both passively as well as actively, has been associated with chronic irritation and discomfort on the eyes, nose and oropharynx.^6^ Since 1964, when a report was published about smoking by the U.S. Department of Health, there already was evidence of cigarette smoking as a factor that worsened and prolonged rhinosinusitis.^7^

Cigarette combustion produces a smoke with more than 4000 noxious components, including gas and particulate substances - among them we have acrolein, formaldehyde, carbon monoxide, nicotine, cotinin; acetaldehyde, phenol and potassium cianide8, and many of these components are provenly toxic to the respiratory epithelium.^9^

One of the possible explanations for cigarette smoke participating in the pathophysiology of CR is based on mucociliary transport alterations. Oral^10^ as well as nasal inhalation^11^ of cigarette smoke causes a deep reduction in mucociliary transport in vivo. Agius et al.^12^ showed that cotinin, a toxic metabolite of nicotine is capable of significantly reducing the cilliary beat of epithelial cells in vitro. In 2009, Cohen et al., also reported on the exposure of epithelia cell cultures to the particulate phase of cigarette smoke reducing cilliary beat increase when stimulated.^13^ Besides in vitro evidence, the exposure to cigarette smoke also harms mucociliary transport in humans, both in acute exposure^14^ as well as in its chronic counterpart.^15^,^16^

Cigarette smoking is also associated with profound changes in mucous production mechanisms. Chronic exposure to this smoke causes metaplastic alterations to the respiratory mucosa with an increase in the number and size of goblet cells and consequent increase in upper airway secretion.^17^,^18^ Cohen et al.^13^ and Kreindler et al.^19^ also showed in vitro that exposure to cigarette smoke inhibits chloride transport in epithelial cells, causing physiological alterations similar to those found in patients with cystic fibrosis.

Besides functional alterations, cigarette smoke causes important structural alterations to the respiratory epithelium. Different studies have shown that cigarette smoke causes a reduction in cell viability and induction of apoptosis in respiratory hair cells^20^, opposite mitogenic effects or pro-apoptotic depending on the cigarette smoke concentration^21^ or even an impairment on epithelial regeneration upon injury.^22^ Animal studies have shown that chronic and intermittent exposure to cigarette smoke cause morphological alterations to the epithelium of the entire respiratory tract, from hyperplasia in lower concentrations, all the way to loss of cilia and metaplasia with keratinization in higher concentrations, and also submucosal thickening and inflammation with neutrofilic and mononuclear inflammatory cells infiltrate.^23^ Also, Hamm et al.^24^ showed that the cigarette-induced respiratory mucosa inflammatory reaction persists even after seven months of recovery. Studies carried out with smokers have confirmed these findings in animal models, showing alterations in the respiratory epithelium as an enlargement of the naked epithelium,^25^ a greater prevalence of hyperplasia and cell atypias^26^ and ultrastructural cilia abnormalities^27^.

**Figure 1:**
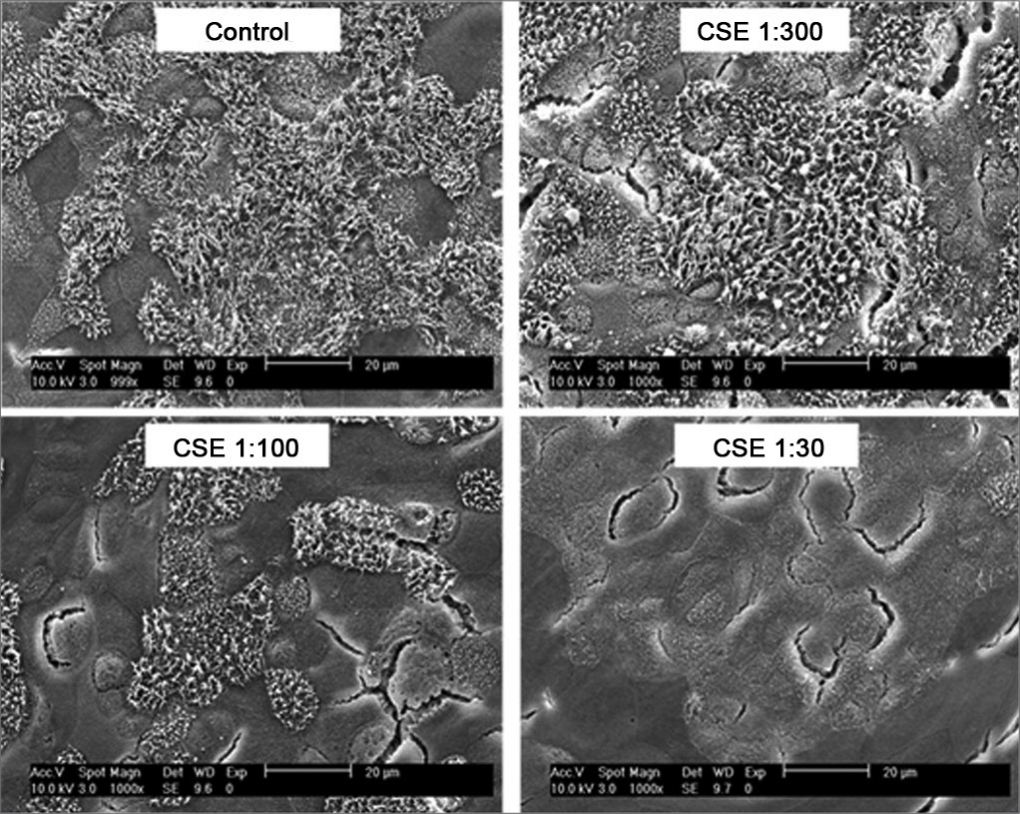
Scanning electron microscopy photographs showing the dose-dependent effect of cigarette smoke on the percentage of ciliated area after exposure to reducing dilutions of Cigarette Smoke Extract (CSE).

**Figure 2:**
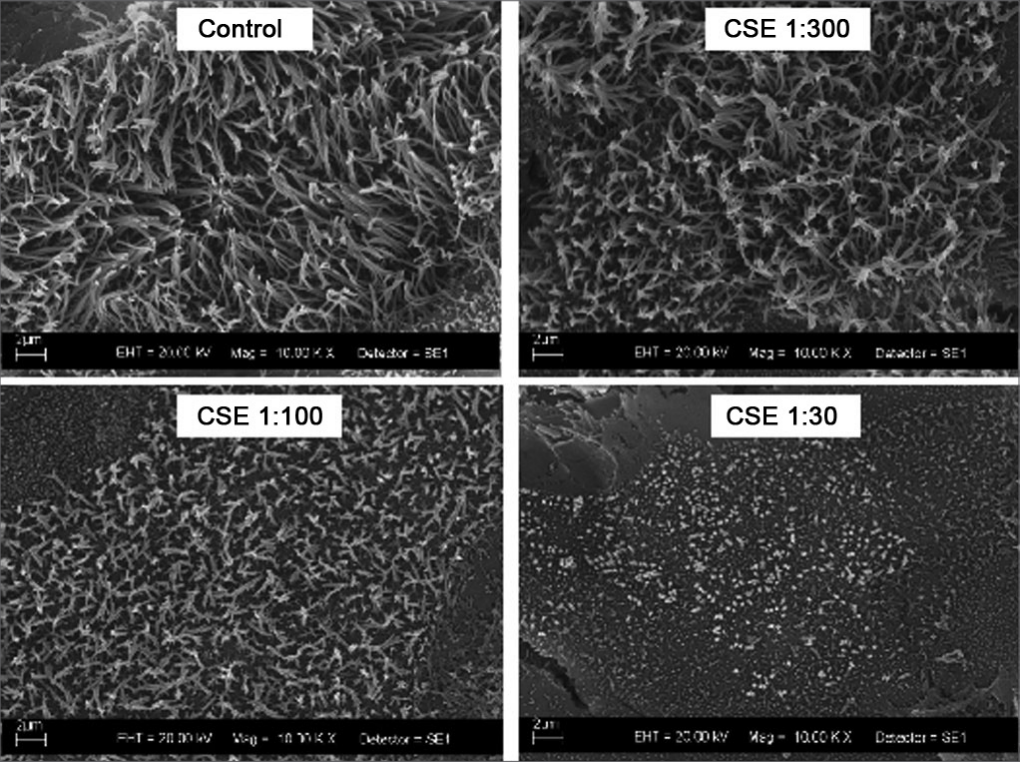
Scanning electron microscopy photographs showing the dose-dependent effect of cigarette smoke on cilia size after exposure to decreasing dilutions of the Cigarette Smoke Extract (CSE).

Besides the alterations caused on the differentiated tissues, Tamashiro et al.^28^ showed that the cigarette smoke has a negative impact on the ciliogenesis process in a dose-dependent fashion to the respiratory epithelium in the maturation and differentiation phases. Using respiratory epithelium cell cultures obtained from the nasal septum of mice, they showed that the respiratory epithelium exposure to the particulate phase as well as the gas phase of cigarette smoke causes a significant reduction in the percentage of cilia development (Figure 1), as well as reduction in their size (Figure 2). Therefore, these authors suggest that there must be more than one toxic substance in the cigarette smoke, particulate and gaseous phases, involved in ciliogenesis block.

2) Evidence of an Association Between Smoking and Chronic Rhinosinusitis

Considering that one of the endpoints of CR is the stasis of nasosinusal secretions secondary to a reduction in mucociliary transport, the alterations caused to the respiratory epithelium would justify a causal relationship of CR with smoking. Notwithstanding, despite the plausible pathophysiological relations, there is little clinical evidence proving this association between smoking and CR.

Lieu and Feinstein (2000)^29^, doing a survey with the American population, observed that smokers had a higher prevalence of chronic or recurrent rhinosinusitis when compared to people who never smoked or who quit smoking. When they compared the relative risk between the groups, this study showed a risk 14% higher for smokers having chronic or recurrent rhinosinusitis, especially those who smoked more than 10 cigarettes per day. Under the viewpoint of the number of cigarettes necessary to cause an effect, we would need 62 active smokers in order to increase in one extra case of chronic rhinosinusitis. Similarly, Chen et al.^30^ did a cross-sectional study between the years of 1996 and 1997 assessing 73,364 Canadian individuals. In such study they reported that smoking was associated to a higher prevalence of chronic rhinosinusitis, both in men and women. Despite a questionable reliability in both studies because they were based on a diagnostic evaluation from a questionnaire, these were the only populational evaluations so far that have identified risk factors for the development of chronic rhinosinusitis.

Other authors have also investigated the influence of cigarette smoke exposure in the postoperative recovery after endoscopic surgery of the paranasal sinuses. In a study with long follow up, Kennedy^31^ showed that smoking was one of the most important factors which led to the need of a second treatment for recurrence. Other studies in adults have also shown that smoking has a negative impact on the postoperative outcome of nasosinusal endoscopic surgery when assessed under the viewpoint of symptoms, quality of life scores, endoscopic analysis and CT-scan findings.^32^,^33^,^34^

In the pediatric population, Kim et al.^35^, retrospectively assessed 97 patients submitted to paranasal sinuses surgery, and showed that children exposed to cigarette smoke had a worse postoperative outcome based on endoscopic analysis. Although the authors included children with asthma (4.1%) in their study, under multivariate analysis they concluded that asthma did not impact the postoperative success of paranasal sinuses endoscopic surgery (p=0.381). By the same token, Ramadan and Hinerman^36^ also showed that the children passively exposed to cigarette smoke had lower improvement rates (70%) when compared to non-exposed children (90%) one year after endoscopic surgery for CR. In this study, Ramadan & Hinerman excluded from the study those children with cystic fibrosis or immunodeficiency and the ones submitted to previous endoscopic surgery.

Another pathophysiological possibility of the association between smoking and CR would be the propensity to bacterial infection on the respiratory epithelium. Ertel et al.^37^ showed that the respiratory tracts of smokers were preferentially colonized by Gram negative bacilli. In this same study, the authors showed that this greater colonization is due to a greater resistance of Gram-negative bacteria to cigarette smoke when compared to Grampositive.^37^ Chronic exposure to cigarette smoke is also capable of increasing the adhesion of these bacteria to epithelial cells, possibly for altering the characteristics of the mucosal surface potentializing the binding of pathogenic bacteria.^38^,^39^ This greater bacteria-epithelium interaction causes an increase in inflammatory reaction on the upper airways by mechanisms that do not depend on toxins.^40^ Still, Tamashiro et al., in 2009, showed that the exposure to high concentrations of cigarette smoke stimulates the in vitro formation of pathogenic bacterial biofilms obtained from patients with CR.^41^

## FINAL REMARKS

Although there is data strengthening a link between smoking and CS, these studies point out that there must be an important individual susceptibility to cigarette smoke response insofar as the development of CS is concerned. At any rate, considering that the main objective of any treatment for patients with CS is to reestablish normal function of the nasosinusal mucosa and the evidence that cigarette smoking impairs mucociliary transport, reducing cilia beat, the ciliogenesis or epithelial regeneration process, a proper education for these patients towards interrupting cigarette smoking as well as reinforcing campaigns against smoking are extremely important to control this disease that has a major socio-economic impact.

## References

[1] Organização Mundial da Saúde, 2008 http://www.who.int/tobacco/mpower/en/acessado em 11 de julho de 2009.

[2] VIGITEL Brasil 2006: Vigilância de Fatores de Risco e Proteção para Doenças Crônicas por inquérito telefônico. Ministério da Saúde. http://portal.saude.gov.br/portal/arquivos/pdf/relatorio_vigitel_2006_marco_2007.pdf). Acessado em 11 de julho de 2009.

[3] Small B (2003). American Academy of Allergy, Asthma and Immunology. Sinusitis: more than just an infection. Asthma Allergy Advoc..

[4] Ray NF, Baraniuk JN, Thamer M, Rinehart CS, Gergen PJ, Kaliner M, Josephs S, Pung YH (1999). Healthcare expenditures for sinusitis in 1996: Contributions of asthma, rhinitis, and other airway disorders. J Allergy Clin Immunol..

[5] Calderón-Garcidueñas L, Valencia-Salazar G, Rodríguez-Alcaraz A, Gambling TM, García R, Osnaya N (2001). Ultrastructural nasal pathology in children chronically and sequentially exposed to air pollutants. Am J Respir Cell Mol Biol..

[6] U.S. Departament of Health and Human Services (1986). The health consequences of involuntary smoking. A report of the Surgeon General, 1986.

[7] Smoking and Health: (1964). Report of the Advisory Committe to the Surgeon General of the Public Health Service.

[9] Dalhamm T (1970). In vivo and in vitro ciliotoxic effects of tobacco smoke. Arch Environ Health..

[10] Ogino S, Nose M, Irifune M, Kikumori H, Igarashi T (1993). Nasal mucociliary in patients with upper and lower respiratory diseases. ORL J Otorhinolaryngol Relat Spec..

[11] Chetan S (1993). Nasal mucociliary clearance in snuff users. J Laryngol Otol..

[12] Agius AM, Wake M, Pahor AL, Smallman LA (1995). Smoking and middle ear ciliary beat frequency in otitis media with effusion. Acta Otolaryngol. (Stockh).

[13] Cohen NA, Zhang S, Sharp DB, Tamashiro E, Chen B, Sorscher EJ (2009). Cigarette smoke condensate inhibits transepithelial chloride transport and ciliary beat frequency. Laryngoscope.

[14] Bascom R, Kesavanathan J, Fitzgerald TK, Cheng KH, Swift DL (1995). Sidestream tobacco smoke exposure acutely alters human nasal mucociliary clearance. Environ Health Perspect..

[15] Stanley PJ, Wilson R, Greenstone MA, MacWilliam L, Cole PJ (1986). Effect of cigarette smoking on nasal mucociliary clearance and ciliary beat frequency. Thorax..

[16] Karaman M, Tek A (2009). Deleterious effect of smoking and nasal septal deviation on mucociliary clearance and improvement after septoplasty. Am J Rhinol Allergy..

[17] Mullen JBM, Wright JL, Wiggs BR, Pare PD, Hogg JC (1987). Structure of central airways in current smokers and ex-smokers with and without mucus hypersecretion: relationship to lung function. Thorax..

[18] Wright JL, Lawson LM, Kennedy S, Wiggs B, Hogg JC (1984). The detection of small airways disease. Am Rev Respir Dis..

[19] Kreindler JL, Jackson AD, Kemp PA, Bridges RJ, Danahay H (2005). Inhibition of chloride secretion in human bronchial epithelial cells by cigarette smoke extract. Am J Physiol Lung Cell Mol Physiol..

[20] Lan MY, Ho CY, Lee TC, Yang AH (2007). Cigarette smoke extract induces cytotoxicity on human nasal epithelial cells. Am J Rhinol..

[21] Luppi F, Aarbiou J, Van Wetering S, Rahman I, De Boer WI, Rabe KF (2005). Effects of cigarette smoke condensate on proliferation and wound closure of bronchial epithelial cells in vitro: role of glutathione. Resp Res..

[22] Van Winkle LS, Evans MJ, Brown CD, Willits NH, Pinkerton KE, Plopper CG (2001). Prior exposure to aged and diluted sidestream cigarette smoke impairs bronchiolar injury and repair. Toxicol Sci..

[23] Gaworski CL, Dozier MM, Eldridge SR, Morrisey R, Rajendran N, Gerhart JM (1998). Cigarette smoke vapor-phase effects in the rat upper respiratory tract. Inhalation Toxicol..

[24] Hamm JT, Yee S, Rajendran N, Morrissey RL, Richter SJ, Misra M (2007). Histological alterations in male A/J mice following nose-only exposure to tobacco smoke. Inhalation Toxicol..

[25] Wanner A, Salathe M, O'Riordan TG (1996). Mucociliary clearance in the airways. Am J Respir Crit Care Med..

[26] Auerback O, Stout AP, Hammond EC, Garfinkel L (1961). Changes in bronchial epithelium in relation to cigarette smoking and in relation to lung cancer. N Engl J Med..

[27] Verra F, Escudier E, Lebargy F, Bernaudin JF, De Cremoux H, Bignon J (1995). Ciliary abnormalities in bronchial epithelium of smokers, ex-smokers, and nonsmokers. Am J Respir Crit Care Med..

[28] Tamashiro E, Xiong G, Anselmo-Lima WT, Kriendler J, Palmer JN, Cohen NA. Cigarette smoke exposure impairs epithelial respiratory ciliogenesis. Am J Rhinol Allergy. 23(2):117–22. 10.2500/ajra.2009.23.3280 19401033

[29] Lieu JE, Feinstein AR (2000). Confirmations and surprises in the association of tobacco use with sinusitis. Arch Otolaryngol Head Neck Surg..

[30] Chen Y, Dales R, Lin M (2003). The epidemiology of chronic rhinosinusitis in Canadians. Laryngoscope..

[31] Kenndey DW (1992). Prognostic factors, outcomes and staging in ethmoid sinus surgery. Laryngoscope..

[32] Briggs RD, Wright ST, Cordes S, Calhoun KH (2004). Smoking in chronic rhinosinusitis: a predictor of poor long-term outcome after endoscopic sinus surgery. Laryngoscope..

[33] Smith TL, Mendolia-Loffredo S, Loehrl TA, Sparapani R, Laud PW, Nattinger AB (2005). Predictive factors and outcomes in endoscopic sinus surgery for chronic rhinosinusitis. Laryngoscope..

[34] Sobol SE, Wright ED, Frenkiel S (1998). One-year outcome analysis of functional endoscopic sinus surgery for chronic sinusitis. J Otolaryngol..

[35] Kim HY, Dhong HJ, Chung SK, Chung YJ, Min JY (2005). Prognostic factors of pediatric endoscopic sinus surgery. Int J Pediatr Otorhinolaryngol..

[36] Ramadan HH, Hinerman RA (2002). Smoke exposure and outcome of endoscopic sinus surgery in children. Otol Head Neck Surg..

[37] Ertel A, Eng R, Smith SM (1991). The differential effect of cigarette smoke on the growth of bacteria found in humans. Chest..

[38] Ozlu T, Celik I, Oztuna F, Bülbül Y, Ozsu S (2008). Streptococcus pneumoniae adherence in rats under different degrees and durations of cigarette smoke. Respiration..

[39] El-Ahmer OR, Essery SD, Saadi AT, Raza MW, Ogilvie MM, Weir DM, Blackwell CC (1999). The effect of cigarette smoke on adherence of respiratory pathogens to buccal epithelial cells. FESS Immunol Med Microbiol..

[40] Drannik AG, Pouladi MA, Robbins CS, Goncharova SI, Kianpour S, Stämpfli MR (2004). Impact of cigarette smoke on clearance and inflammation after Pseudomonas aeruginosa infection. Am J Respir Crit Care Med..

[41] Tamashiro E. Efeitos da exposição à fumaça de cigarro sobre a ciliogênese e formação de biofilmes bacterianos no epitélio respiratório [Tese de Doutorado] - Faculdade de Medicina de Ribeirão Preto -Universidade de São Paulo, 2009.

